# Five-lipoxygenase-activating protein-mediated CYLD attenuation is a candidate driver in hepatic malignant lesion

**DOI:** 10.3389/fonc.2022.912881

**Published:** 2022-08-01

**Authors:** Kun-kai Su, Xue-hua Zheng, Christian Bréchot, Xiao-ping Zheng, Dan-hua Zhu, Rong Huang, Yan-hong Zhang, Jing-jing Tao, Yi-jia Lou, Lan-juan Li

**Affiliations:** ^1^ State Key Laboratory for Diagnosis and Treatment of Infectious Diseases, First Affiliated Hospital, School of Medicine, Zhejiang University, Hangzhou, China; ^2^ National Clinical Research Center for Infectious Diseases, First Affiliated Hospital, School of Medicine, Zhejiang University, Hangzhou, China; ^3^ Institute of Pharmacology and Toxicology, College of Pharmaceutical Sciences, Zhejiang University, Hangzhou, China; ^4^ Department of Pharmacology, Shengjing Hospital, China Medical University, Shenyang, China; ^5^ Institut Pasteur, Paris, France; ^6^ Department of Pathology, Shulan (Hangzhou) Hospital, Hangzhou, China

**Keywords:** hepatocellular carcinoma, lipid pro-inflammatory mediators, 5-lipoxygenase pathway, median survival time, candidate biomarker

## Abstract

Hepatocellular carcinoma (HCC) is an inflammation-associated cancer. However, the lipid pro-inflammatory mediators have only been seldom investigated in HCC pathogenesis. Cylindromatosis (CYLD) attenuation is involved in hepatocarcinogenesis. Here, we aimed to evaluate the significance of hepatic lipid pro-inflammatory metabolites of arachidonate-affected CYLD expression *via* the 5-lipoxygenase (5-LO) pathway. Resection liver tissues from HCC patients or donors were evaluated for the correlation of 5-LO/cysteinyl leukotrienes (CysLTs) signaling to the expression of CYLD. The impact of functional components in 5-LO/CysLTs cascade on survival of HCC patients was subsequently assessed. Both livers from canines, a preponderant animal for cancer research, and genetic-modified human HCC cells treated with hepatocarcinogen aristolochic acid I (AAI) were further used to reveal the possible relevance between 5-LO pathway activation and CYLD suppression. Five-LO-activating protein (FLAP), an essential partner of 5-LO, was significantly overexpressed and was parallel to CYLD depression, CD34 neovascular localization, and high Ki-67 expression in the resection tissues from HCC patients. Importantly, high hepatic *FLAP* transcription markedly shortened the median survival time of HCC patients after surgical resection. In the livers of AAI-treated canines, FLAP overexpression was parallel to enhanced CysLTs contents and the simultaneous attenuation of CYLD. Moreover, knock-in *FLAP* significantly diminished the expression of CYLD in AAI-treated human HCC cells. In summary, the hepatic FLAP/CysLTs axis is a crucial suppressor of CYLD in HCC pathogenesis, which highlights a novel mechanism in hepatocarcinogenesis and progression. FLAP therefore can be explored for the early HCC detection and a target of anti-HCC therapy.

## Introduction

**Figure 1:**
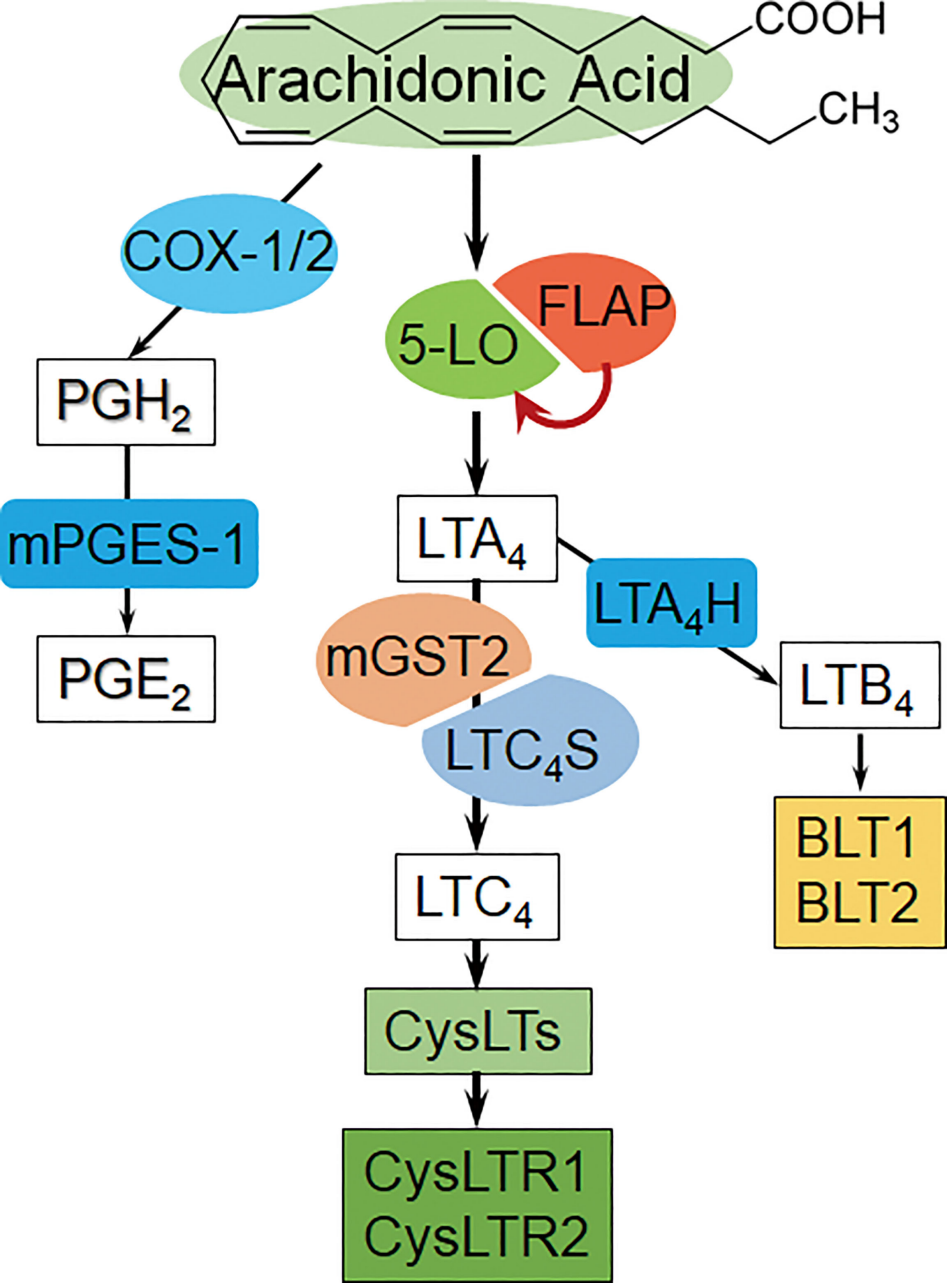
The overview diagram of arachidonic acid-derived metabolite pathways. **Left:** COX/PGE_2_ pathway, COX: Cyclooxygenase, mPGES-1: microsomal prostaglandin E synthase-1.. **Middle:** 5-LO/CysLTs cascade and the receptors [AA is catalyzed by 5-LO/FLAP to form primary metabolite LTA_4_, and then converted to LTC_4_ *via* LTC_4_S (in leukocytes) or mGST2 (in a wide variety of cells); subsequently, LTC_4_ exports from cells, and extracellularly cleaves and sequentially forms CysLTs]. **Right:** LTB_4_ bypass, BLT1/2: LTB_4_ receptor 1/2. (Summarized from the available references).

Hepatocellular carcinoma (HCC) is one of the most lethal malignancies and the second leading cause of cancer death worldwide (1–3). HCC surveillance is associated with early tumor detection and improved survival in patients with liver diseases (4). To date, it is not fully elucidated how initial molecular events and signaling involve the onset and progression of HCC. HCC is defined as an inflammation-associated cancer (5). Hepatocarcinogenesis and progression may evolve from the inflammation. However, the lipid pro-inflammatory mediators have only been seldom investigated in HCC pathogenesis. Cysteinyl leukotrienes (CysLTs), including LTC_4_, LTD_4_, and LTE_4_, are lipid-signaling molecules that mediate both acute and chronic inflammation (6). CysLTs constitute the major products of arachidonic acid (AA) metabolism *via* the 5-lipoxygenase (5-LO) pathway (7, 8), forming 5-LO/CysLTs cascade. The integral membrane protein 5-LO-activating protein (FLAP) is an essential partner of 5-LO for the first step in LTs synthesis (9, 10); it selectively transfers AA to 5-LO and enables the sequential oxygenation of AA (11). Any factor inducing FLAP expression can lead to the enhancement of CysLTs synthesis. The primary metabolite LTA_4_ is further converted to LTC_4_ *via* LTC_4_ synthase (LTC_4_S) or its isoenzyme, membrane-embedded microsomal glutathione-*S*-transferase 2 (mGST2) (12–14). CysLTs bind at different affinities to two classical G-protein-coupled receptors: CysLTR1 and CysLTR2 (8, 15) (**Figure 1**, an overview from the available references). Activation of CysLTRs exerts downstream effects, including chemokine production, immune cell activation, and tissue inflammation (16). Cancer cells may utilize the 5-LO pathway to interact with tumor microenvironment during the development and progression of a tumor (8, 17). Chemical-induced liver damage in animal models displays the 5-LO-pathway activation and CysLTs content elevation (7, 18, 19). Hepatitis B virus X protein enhances proliferation of hepatoma cells *via* AA metabolism-associated pathways *in vitro* (20). However, the underlying mechanism by which CysLTs contribute to HCC pathogenesis is so far not well documented. Whether 5-LO/CysLTs cascade also takes a critical role in promoting hepatocarcinogenesis and development is still elusive.

Cylindromatosis (CYLD), a deubiquitination enzyme, negatively regulates cancer progression (21). Livers in CYLD-deficient mice display spontaneous Kupffer cell activation, inflammatory cell infiltration, nuclear factor (NF)-κB activation, oncoprotein c-Myc expression, and potentiated tumor development induced by diethylnitrosamine (22, 23). CysLTs can initiate NF-κB constitutively active in up to 80% of colorectal tumors (24, 25). However, it is still unclear whether 5-LO/CysLTs cascade is required for CYLD suppression. Understanding the relevance of the 5-LO pathway to the CYLD expression, and the signal cooperativity might provide a deeper look into the mechanism in hepatocarcinogenesis and HCC development.

Short-term aristolochic acid I (AAI) exposure leads to NF-κB signaling activation and c-Myc expression in kidneys (AAI-DNA adduct target) and livers (AAI-DNA adduct nontarget) of human *TP53* knock-in mice (26). Owing to the inherent biological nature of dogs, canine and human share the remarkable similarity in cancers (27, 28). Our previous study confirms that hepatic premalignant lesion appears in canines after a 10-day AAI oral administration. It is featured by the overexpression of c-Myc and oncofetal RNA-binding protein Lin28B, the enhancement of *Il6* [interleukin (IL)-6] transcription, and the increase of supernatant IL-6 level (29). The premalignant node involves dysregulations of microRNAs, as well as activation of IL-6 receptor, p-STAT3, and NF-κB (29). Therefore, it is extremely valuable to further explore whether 5-LO/CysLTs cascade is associated with AAI-induced hepatocarcinogenesis and involved in CYLD suppression.

In the present study, we report that FLAP highly expresses in the resection tissues from HCC patients. Furthermore, high transcription of *FLAP* in resection tissue significantly shortens the median survival time (MST) of HCC patients after surgical resection. Using an AAI-treated canine model and human HCC cell lines, we further highlight the potential role of the hepatic FLAP/CysLTs cascade in CYLD attenuation. This finding provides the hopeful target for early HCC detection and anti-HCC drug development.

## Materials and methods

### Human subjects

Human liver samples were obtained from HCC patients undergoing surgical resection or liver transplantation, and healthy donors for liver transplantation in the form of histologic section at Shulan (Hangzhou) Hospital. Protocols for patient tissue section usage were reviewed and approved by the Ethics Committees of Shulan (Hangzhou) Hospital (№ 20170207). Written informed consent was obtained. All experiments were done in accordance with the governmental and institutional guidelines of the ICH-GCP (according to the principles of the Declaration of Helsinki). Histopathological grading was performed at the Department of Pathology in Shulan (Hangzhou) hospitals. All the participants are anonymized and summarized in **Supplementary Table 1**.

### Bioinformatics analysis of gene transcriptions

MRNA sequencing data of HCC tissues and clinical follow-up record of the HCC cohort were downloaded from The Cancer Genome Atlas (TCGA) in accordance with the data usage policy. MRNA expression of the indicated genes was retrieved (30). Patients were allocated into dichotomized subgroups based on processed expression for the survival analysis. Kaplan–Meier plots were used for comparison of survival curves, and log-rank tests were applied for *p*-value calculation. The correlation between the transcription level of *FLAP* in resection tissue and the MST of HCC patients after surgical resection was evaluated.

### Animal experimentation

Ten-month-old male beagle canines were purchased from the Nanjing Anlimo Tech. Co. Ltd. (Nanjing, China) and maintained in a specific pathogen-free environment, since they were a preponderant animal for cancer research (27, 28). The protocols were approved by the Animal Care and Use Committee of Zhejiang University (№ ZJU2009108012Y), and all procedures were performed in accordance with the institutional guidelines.

AAI (purity >98%, HPLC, Delta) was mixed with filler and filled into capsules. Canines were randomly assigned into two groups and given capsules with AAI filler (3 mg/kg/day) or control filler only for 10 days (29). Canines were sacrificed 11 days after the initiation of the treatment. Livers were excised immediately after sacrifice. Part of the liver was fixed in 4% (wt/vol) neutral buffered formalin (pH 7.4) and embedded in paraffin for histologic analyses. The remaining liver tissues were immediately snap-frozen in liquid nitrogen and kept at −80°C until use.

### Antibodies

Immunohistochemistry: Anti-FLAP (sc-28815), mGST2 (sc-65130), and LTC_4_S (sc-22564) were purchased from Santa Cruz Biotechnology (California, USA). CysLTR2 (ab32536) was purchased from Multisciences Biotech (Hangzhou, China). CYLD (#8462) was purchased from Cell Signaling Technology (Danvers, USA). 4′,6′-Diamidino-2-phenylindole (DAPI) (ZLI-9557) was purchased from Zhongshan Jinqiao Biotechnology (Beijing, China). Goat Anti-Rabbit IgG Dylight 549 (GAR5492) was purchased from Multisciences Biotech. Goat Anti-Rabbit IgG/Alexa Fluor 488 (ZF-0511) was purchased from Yeasen Biotech.

Western blot: Anti-FLAP, mGST2, LTC_4_S, CysLTR2, and CYLD were the same as mentioned above. 5-LO (sc-8885) was purchased from Santa Cruz Biotechnology. LTB_4_ receptor 1 (BLT1) (ab131041) and BLT2 (ab84600) were purchased from Abcam (Cambridge, England). CysLTR1 (ab32534) was purchased from Multisciences Biotech (Shanghai, China). Antibodies of c-Jun N-terminal kinase (JNK) (#9252), phospho-JNK (p-JNK) (#9251), eukaryotic translation initiation factor 2 alpha (eIF2a) (#2103), p-eIF2a (#3398), 78-kDa glucose-regulated protein (GRP78) (#3177), and 94-kDa Glucose-Regulated Protein (GRP94) (#2104) were purchased from Cell Signaling Technology. Microsomal prostaglandin E synthase-1 (mPGES-1) (#160140) was purchased from Cayman Chemical (Ann Arbor, USA). Β-Actin (Mab1445), GAPDH (Mab5465-040), α-Tubulin (ab36864), β-Tubulin (ab012), and the HRP-conjugated secondary antibodies (goat anti-mouse, LK-GAM007; goat anti-rabbit, LK-GAR007; and rabbit anti-goat, LK-RAG007) were purchased from Multisciences Biotech.

### Histological assessments of liver tissues

Human liver paraffin-embedded consecutive 4-μm-thick sections were used for immunohistochemical analysis. Anti-Ki-67 (IR098) and CD34 (IM034) were purchased from LBP (Guangzhou, China). Canine liver sections were prepared as described previously (29) for hematoxylin and eosin (H&E) or immunohistochemical stains, and consecutive 8-µm-thick frozen sections (cut by a freezing microtome, CM1950, Leica, Wetzlar, Germany) were used for immunofluorescent staining. Immunofluorescence analysis was performed using a confocal microscope (TCS SP8 MP, Leica) or a microscope (Nikon Eclipse 80i). Both human and canine liver sections were stained with the indicated antibodies to distinguish the immunohistochemical or immunofluorescence changes in expression and distribution *in situ*. For Ki-67 tissue evaluation, sections were graded based on the percentage of Ki-67 positively stained nuclei, using the range 0%–100%.

### Western blot analysis and ELISA

Western blot assay was performed as described previously (29). Briefly, samples were homogenized with RIPA buffer (P0013K, Beyotime, Shanghai, China) for protein extraction. Whole blotting gel quantified with a fluorescence scanner imaging system (Bio-Rad, Hercules, California, USA) and densitometry was performed using ImageJ software (https://imagej.nih.gov/ij, NIH, Bethesda, MD, USA).

Supernatant containing CysLTs was extracted from liver tissue homogenate of canines (1:10 wt/vol, 50 mmol/L Tris-HCl, pH 7.5). CysLTs level was measured by ELISA Kit (Cayman, USA) on a DTX 880 Multimode Detector (Beckman Coulter, USA).

### Real-time qRT-PCR analysis

Real-time qRT-PCR analysis was conducted according to the protocol (31). Briefly, total RNAs from human HCC cell Lines and canine livers were isolated with TRIzol reagent (Gibco BRL, USA). After 2 min of initial denaturation at 95°C, amplification used 40 cycles following 15 s at 95°C and 1 min at 60°C. The primer sequences (5’-3’) for *FLAP*, *Lta4h* (LTA_4_ hydrolase), and *GAPDH* (in human HCC cells) were documented in **Supplementary Table 2**.

### Cell culture and genetic handling of *FLAP* in human HCC cells

The human HCC cell lines HepG2, Hep3B2.1-7 (Hep3B), and PLC/PRF/5 were purchased from Shanghai Institute of Cell Bank, Chinese Academy of Sciences (№ 22008). Cells were maintained in DMEM (12800-017, Life Technologies, USA) supplemented with 10% fetal bovine serum (FBS; 10099-141, Gibco, Germany).

Overexpression of *FLAP* in HepG2 or Hep3B cells was achieved by lentiviral infection. Lentiviruses (lv) were constructed, concentrated, and purified by Genechem (Shanghai, China). HepG2 or Hep3B cells were infected with lv-*Control* (lv-*Con*) or lv-*FLAP* (HepG2^lv-^ *^Con^*, HepG2^lv-^ *^FLAP^*, Hep3B ^lv-^ *^Con^*, and Hep3B ^lv-^ *^FLAP^*) (MOI = 10), and then selected for 3 days in the presence of 2.5 μg/ml puromycin (Gibco, Germany). Knockdown of *FLAP* in HepG2 or Hep3B cells was conducted by short hairpin RNA (shRNA). Sh-*Con* and sh-*FLAP* plasmids (Sangon Biotech, Shanghai, China) were transfected with Lipofectamine 2000 transfection agent (HepG2^sh-^ *^Con,^* HepG2^sh-^ *^FLAP^*, Hep3B^sh-^ *^Con^*, and Hep3B^sh-^ *^FLAP^*) (Invitrogen, Life Technologies) according to the manufacturer’s instructions. Sequences for shRNA are shown in **Supplementary Table 3**. Lv-RNA or shRNA interfering efficiency was validated by qRT-PCR.

All the knock-in or knock-down processed cells together with wild-type (WT) HCC cell lines were further incubated with AAI (final concentration, 1.25 μM) for another 7 days. Cells were harvested on day 10 after initiation of infection or transfection. The expression of FLAP in HepG2 cells was examined by flow cytometry according to commercial instructions. Finally, cells were analyzed by a BD FACSCount II Flow Cytometer (BD Biosciences, San Jose, USA) or a microscope (Nikon Eclipse 80i) after immunofluorescent staining. Stunning (for Hep3B cells).

### Statistical analysis

Pairwise comparisons between continuous data were analyzed using an unpaired two-tailed Student *t-*test, and multiple comparisons were analyzed by one-way ANOVA. All data were expressed as mean ± SD, and *p* < 0.05 was considered statistically significant. Significant *p-*values are indicated by asterisks in the individual figures.

### Results

#### FLAP overexpression is paralleled to CYLD suppression in resection tissues from HCC patients

**Figure 2:**
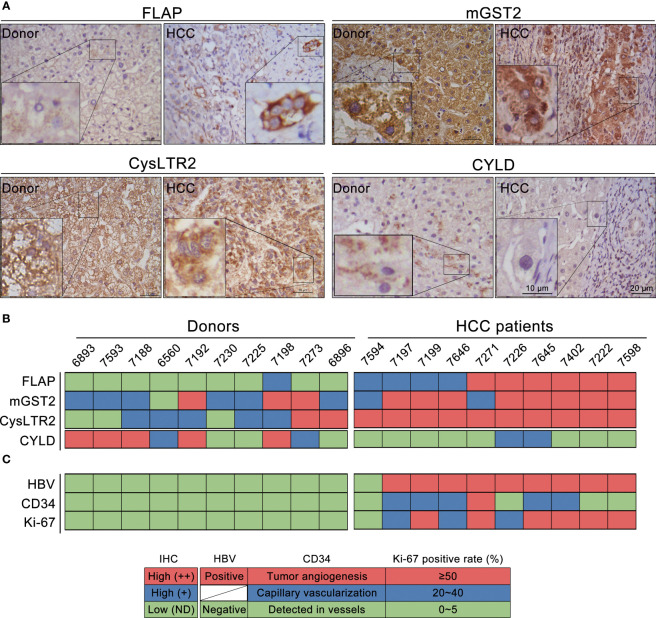
Overexpression of FLAP correlates to CYLD suppression in resection tissues of HCC patients. **(A)** Representative immunohistochemistry (IHC) images of FLAP, MGST2, CysLTR2, and CYLD in each subject using the indicated antibodies. Scale bar = 20 μm, bar = 10 μm (magnification). **(B)** The expression levels of FLAP, mGST2, CysLTR2, and CYLD were clustered. Patient and donor codes are indicated. Grades of the indicated markers (Red: **++** = brown, Blue: **+** = golden, Green: ND = not detected). **(C)** The expression levels of CD34 localization and positive rate (%) for Ki-67 were clustered. Patient and donor codes are matched with each other. For each analysis, *n* = 10/per group.

We explored the impact of functional components in CysLTs biosynthesis on CYLD expression in the resection tissues from 10 HCC patients. CysLTs biosynthesis exhibits AA metabolism through the 5-LO pathway. To investigate the involvement of FLAP in the progression of HCC, we examined its expression and localization in the resection tissues. Patients’ characteristics, concerning etiology, gender, age, and tumor stage, were summarized (**Supplementary Table 1**). Immunohistochemical staining revealed that the FLAP level was considerably higher in the resection tissues from HCC patients as compared to the samples from donor controls (**Figure 2A**, upper left panel). All the HCC resection tissues displayed a higher FLAP expression, but only 1 in 10 liver tissues from donors expressed marginal FLAP (**Figure 2B**). In contrast, the resection tissues from HCC patients had a markedly lower CYLD expression than the samples from donor controls (**Figure 2A**, bottom right panel). Considering that both mGST2 and CysLTR2 in the 5-LO pathway were basically required in the lipid pro-inflammatory mediators’ signaling transduction, we subsequently observed their abundance, and further clustered all the four molecular events according to their expression grades in resection tissues from HCC patients or donors (**Figures 2A**, **B**). In parallel to high FLAP expression, 8 in 10 resection HCC tissues showed the absence of CYLD expression (**Figure 2B**). As to the two cases with CYLD expression, one showed CYLD nuclear translocation together with a much higher cytosolic CysLTR2 expression within poorly differentiated HCC lesions (**Supplementary Figure 1**, left panel), and the other one displayed slight cytosolic CYLD expression together with a much higher nuclear CysLTR2 expression in paracancerous tissue (**Supplementary Figure 1**, right panel). Furthermore, compared to the ratio of overexpression of mGST2 (3/10) and CysLTR2 (2/10) in donor livers, there are much more overexpression of mGST2 (8/10) and CysLTR2 (10/10) in HCC tissues (**Figure 2B**). According to the clustered results, the hepatic mGST2 and CysLTR2 have a basal expression level under physiological conditions, but their expressions were robustly enhanced in the malignant lesion, depending on the upstream element FLAP level. Strikingly, there was less significance of high expressions of mGST2 and CysLTR2 to CYLD suppression in the livers of donors, implying that mGST2 and CysLTR2 were not prerequisite for inhibiting CYLD expression. Our data strongly suggested a negative correspondence between FLAP overexpression and CYLD attenuation in HCC tissues (**Figure 2B**), suggesting that overexpressed FLAP was a potential hallmark of HCC. We further identified the effects of overexpressed FLAP on HCC invasiveness and development, as evidenced by evaluating the downstream effectors. We found that CD34, a marker of hematopoietic stem cells, and Ki-67, a marker of cell proliferation, were highly expressed in resection tissues of HCC in contrast to donors (**Figure 2C**). Of 10 resection tissues of HCC, 6 showed CD34 neovascular localization (including tumor angiogenesis), and 9 of 10 resection tissues of HCC showed Ki-67 overexpression (**Figure 2C** and **Supplementary Table 1**). Importantly, the HCC tissues that exhibited FLAP overexpression with ++ grades (≥50% positive rate) also showed Ki-67 expression with ++ grades. In contrast, the donor livers without FLAP expression showed neither CD34 nor Ki-67 expression (**Figure 2C**). These data implied that overexpressed FLAP was correlated with CYLD suppression, and involved in neovascularization and tumor cell proliferation in HCC tissues.

### Enhanced *FLAP* transcription shortens the median survival time of HCC patients after surgical resection

**Figure 3:**
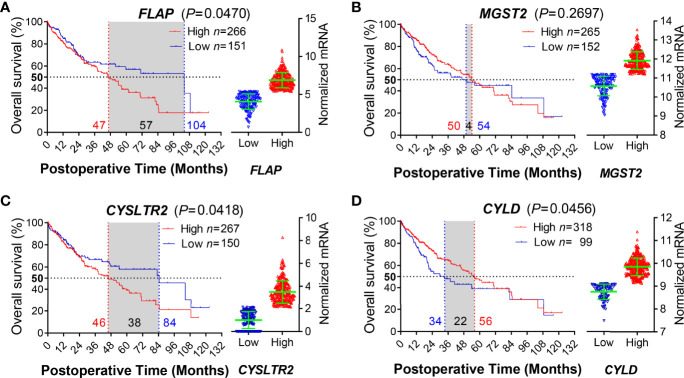
Enhanced hepatic *FLAP* mRNA shortens median survival time of HCC patients after surgical resection. Data for either HCC patients after surgical resection or liver transplantation from the TCGA Research Network (http://cancergenome.nih.gov/) were eligible for survival analysis. **(A–D) Left panels:** Kaplan–Meier survival curves in coordinate axis for comparing survival rates between high- and low-gene transcription subgroups from the HCC cohort. Horizontal dotted line: 50% survival. Vertical red/blue dotted lines: median survival time (MST). **(A)** *FLAP* mRNA (*p* = 0.0470). **(B)** *mGST2* mRNA (*p* = 0.2697). **(C)** *CysLTR2* mRNA (*p* = 0.0418). **(D)** *CYLD* mRNA (*p* = 0.0456). **Right panels:** Scattered plots of transcription levels for indicating genes from the HCC cohort. Student unpaired *t-*test was used to compare groups and *p-*value less than 0.05 was considered as statistically significant.

To support the lines of evidence for the role of FLAP on the clinical findings described above, we took advantage of the long-term clinical follow-up information provided by TCGA for bioinformatics analysis. We obtained a 417-patient HCC cohort, which was well documented with survival information. We subsequently analyzed the relationships between the transcription levels of hepatic *FLAP*, *mGST2, CysLTR2* and *CYLD* and the MST in HCC patients after surgical resection, respectively. Notably, MST was only 47 months for high *FLAP* mRNA in HCC patients (*p* = 0.04), in contrast to 104 months for low *FLAP* mRNA (**Figure 3A**). Conversely, there was only a slightly higher effect between high and low *mGST2* mRNA on MST in HCC patients (*p* = 0.27) after surgical resection (**Figure 3B**). On the other hand, higher *CysLTR2* mRNA significantly shortened MST in HCC patients (*p* = 0.04) after surgical resection (**Figure 3C**). Furthermore, MST was as low as 34 months for low *CYLD* mRNA and 56 months for high *CYLD* mRNA in HCC patients after surgical resection (*p* = 0.04) (**Figure 3D**). Remarkably, the effects of hepatic *FLAP* or *CysLTR2* and *CYLD* transcription on MST also displayed a negative correlation in HCC patients after surgical resection. Obviously, the patients with low *FLAP* mRNA had a much longer survival period (MST, 104 months) than those with low *CysLTR2* mRNA (MST, 84 months), indicating that the transcription level of *FLAP* is one of the most crucial impacts on survival of HCC patients after surgical resection. From the MST point of view, enhanced *FLAP* appeared much earlier than attenuated *CYLD* in HCC patients (**Figures 3A, D**); therefore, FLAP possessed the crucial value to evaluate the progress and recrudescence of HCC patients after surgical resection.

### Hepatic CYLD attenuation and CysLTs increasement after AAI treatment

**Figure 4:**
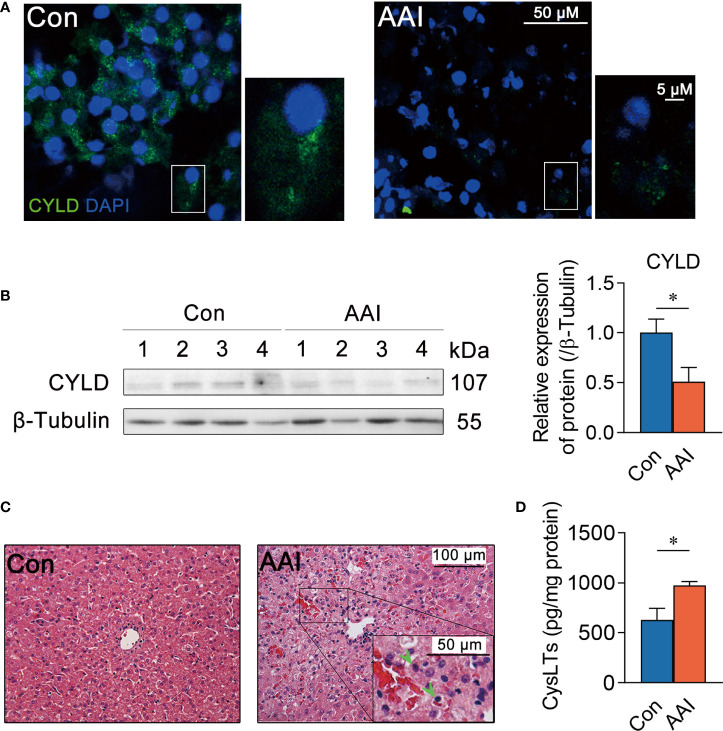
Hepatic CYLD attenuation is parallel to increased CysLTs production after AAI treatment in canines. Liver tissues were obtained from canines after treatment with AAI (3 mg/kg/day) or filler only (negative control, Con) for 10 days. **(A)** Representative photomicrographs of CYLD (green: distribution in cytoplasm, DAPI: nuclei) in liver sections stained by immunofluorescence histochemistry. Bar = 50 μm, bar = 5μm (magnification). **(B)** Immunoblot analysis of hepatic CYLD expressions. β-Tubulin was used as an internal loading control. **(C)** Representative photomicrographs of liver sections stained with H&E (inflammatory cells, arrowheads). Bar = 100 μm, bar = 50 μm (magnification). **(D)** Supernatant CysLTs level in liver tissue homogenate detected by ELISA. Error bars represent the mean value ± SD; Student unpaired *t-*test was used to compare groups, and *p-*value less than 0.05 was considered as statistically significant. *p < 0.05 vs. control. For each analysis, *n* = 4/per group.

To determine whether 5-LO/CysLTs cascade influences CYLD depression, we first assessed the effect of AAI on CYLD expression in livers of canines. Compared to control, both the abundance of CYLD in hepatocytes and the numbers of CYLD-positive hepatocytes by AAI treatment extremely decreased using immunofluorescence staining (**Figure 4A**). Western blot analysis further confirmed that AAI treatment significantly attenuated the expression of CYLD in liver tissues (**Figure 4B**). Next, H&E-stained liver sections displayed inflammatory cell infiltration around the central veins after AAI treatment (**Figure 4C**, **Supplementary Figure 2**), which was similar to the feature that spontaneously appeared in the livers of CYLD-deficient mice (32). It was also well matched with our previous findings of an increase in serum ALT and AST, as well as in *Il6* transcription and supernatant IL-6 level in AAI-treated livers (29). We then further examined the effect of AAI on CysLTs biosynthesis in canine livers. As determined by ELISA, AAI treatment significantly raised the content of CysLTs in liver homogenate supernatant compared to control (**Figure 4D**). These data suggested that CysLTs generation was parallel to CYLD attenuation accompanied by the CYLD-deficient feature of inflammatory cell infiltration around the central veins in AAI-treated canine livers.

### Hepatic FLAP overexpression contributes to CysLTs increase

**Figure 5:**
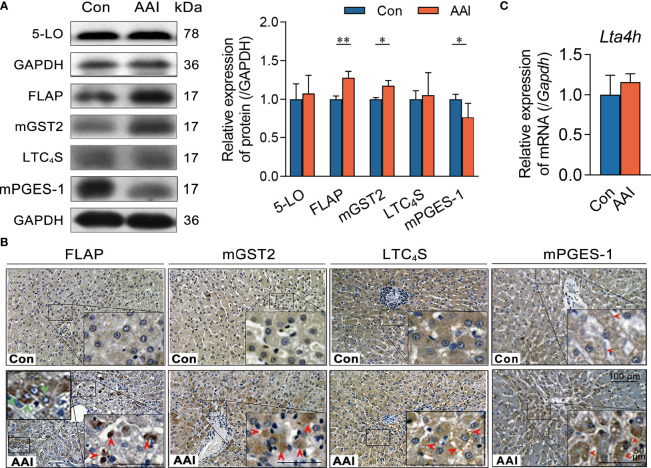
AAI treatment leads to FLAP overexpression in canine livers. Liver tissues were obtained from canines after treatment with AAI (3 mg/kg/day) or filler only (negative control, Con) for 10 days. **(A)** Representative Western blotting of the catalytic proteins in 5-LO/CysLTs cascade and COX pathway. **(B)** Representative photomicrographs with immunohistochemical stains using indicated antibodies in 5-LO/CysLTs cascade and COX pathway. FLAP in hepatocytes (dark brown, green arrowheads), Kupffer cells (dark brown, red arrowheads), mGST2 (light brown, arrowheads), and LTC_4_S (arrowheads) in liver tissues. MPEGS-1 (slight brown round central veins, disappearing in elsewhere). Bar = 100 μm, bar = 50 μm (magnification). **(C)** The transcription of hepatic *Lta4h* was analyzed by real-time quantitative PCR. Error bars represent the mean value ± SD; Student unpaired *t-*test was used to compare groups, and *p-*value less than 0.05 was considered as statistically significant. *p < 0.05, **p < 0.01 vs. control. For each analysis, *n* = 4/per group.

To obtain functional insights into hepatic 5-LO/CysLTs cascade in AAI-induced CYLD suppression, we observed the expression of each component in the 5-LO pathway. Using Western blotting, we evaluated the expressions of four catalytic proteins involved in the LTC_4_ biosynthetic process, and found that FLAP and mGST2 were significantly higher in expression compared to control (*p* < 0.01 and *p* < 0.05, respectively) in AAI-treated livers (**Figure 5A**). In particular, we individually identified the abundance and distribution of each protein involved in LTC_4_ biosynthesis in the liver tissues with immunohistochemical staining. We observed that, compared to control, AAI induced considerable overexpression of FLAP in both hepatocytes and Kupffer cells. MGST2 was highly expressed in hepatocytes, but the expression of LTC_4_S did not change after AAI treatment (**Figure 5B**, the positive or negative control for immunohistochemical analysis saw in **Supplementary Figure 3**). In contrast, AAI failed to increase the transcription of hepatic *Lta4h* at the LTA_4_-derived LTB_4_ step in the 5-LO bypass (**Figure 5C**) (33). Conversely, AAI significantly inhibited the expression of hepatic mPGES-1 (*p* < 0.05) (**Figures 5A, B**), which catalyzes prostaglandin (PG) E_2_ generation at the terminal steps of the cyclooxygenase (COX) pathway. These data indicated that neither the 5-LO/LTB_4_ bypass nor the COX/PGE_2_ pathway contributed to AAI-induced hepatic CYLD suppression. Therefore, AAI-treated livers underwent FLAP/CysLTs cascade-associated pathogenesis.

### Endoplasmic reticulum stress contributes to CysLTs/CysLTR2 signaling transduction

**Figure 6:**
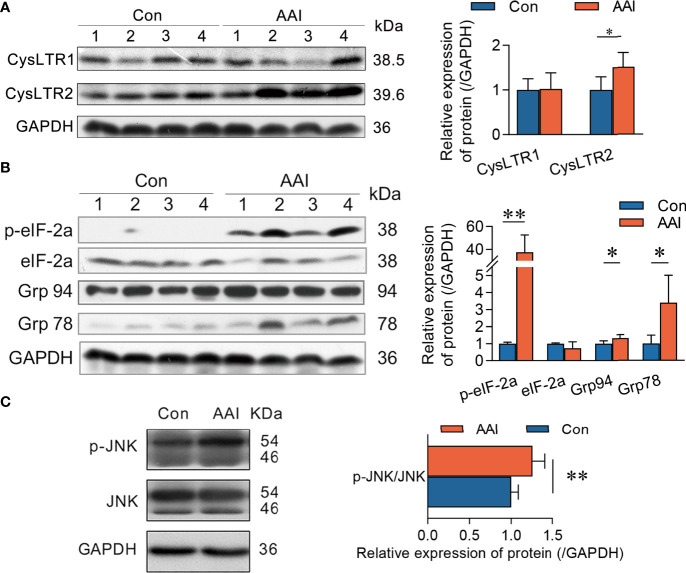
CysLTR2 overexpression along with ER stress and JNK phosphorylation in AAI-treated livers of canines. Liver tissues were obtained from canines after treatment with AAI (3 mg/kg/day) or filler only (negative control, Con) for 10 days. Immunoblot analyses of **(A)** CysLTRs, **(B)** molecular events for ER stress, and **(C)** phosphorylated JNK. GAPDH was used as loading control. Error bars represent the mean value ± SD. Student unpaired *t-*test was used to compare groups, and *p-*value less than 0.05 was considered as statistically significant. *p < 0.05 vs. control. **p < 0.01 vs. control. For each analysis, *n* = 4/per group.

To understand the underlying molecular mechanism of CysLTs associated with CYLD attenuation after AAI treatment, we subsequently focused on the connection of CysLTs/CysLTRs signaling in canine livers. Meanwhile, we also checked endoplasmic reticulum (ER) stress, since ER stress inducers showed the ability to reduce CYLD expression (34). Using Western blotting, we detected the expressions of the hepatic CysLTR1, CysLTR2, BLT1 (the high-affinity receptor of LTB_4_), BLT2, Grp78, and Grp 94 (the early events of ER stress), as well as the phosphorylation of eIF2α (the late event of ER stress) once stimulated with AAI. We observed that, compared to control, AAI significantly upregulated CysLTR2 expression (*p* < 0.05), but did not influence the expressions of CysLTR1 (**Figure 6A**), BLT1, and BLT2 (**Supplementary Figure 4**). These data indicated that, among the hepatic receptors associated with LTs, only CysLTR2 displayed overexpression in response to AAI treatment. Furthermore, AAI increased in both expression of Grp78 (*p* < 0.05) and phosphorylation of eIF2α (*p* < 0.01) (**Figure 6B**), suggesting that a possible relevance existed in ER stress and CysLTR2 activation. We next measured the phosphorylation of JNK, one of the downstream effectors of the CYLD attenuation, and found that JNK was significantly phosphorylated (*p* < 0.01) (**Figure 6C**). Taken together, AAI-induced high expression of CysLTR2 was accompanied by ER stress and phosphorylation of JNK, implying that the ER stress/CysLTR2 axis probably promoted the FLAP/CysLTs/CYLD/p-JNK signaling transduction in AAI-treated livers.

### Knock-in *FLAP* diminishes CYLD expression in human HCC cell lines

**Figure 7:**
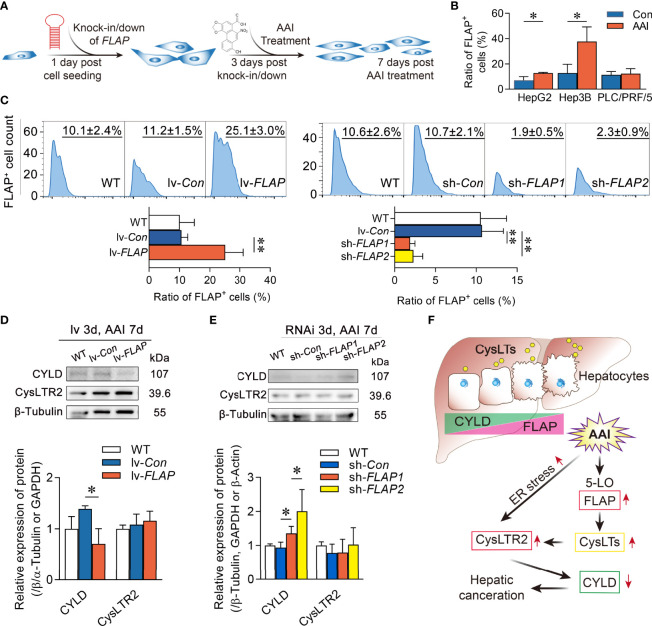
Overexpression of FLAP diminishes CYLD expression in AAI-treated HepG2^lv-^ *^FLAP^* cells. **(A)** Scheme of the experimental procedure. **(B)** FLAP-positive (FLAP^+^) cell ratio in human HCC cell lines HepG2, Hep3B, and PLC/PRF/5 in response to AAI by flow cytometry assay, respectively. WT HCC cells without AAI treatment were used as control. **(C)** FLAP-positive (FLAP^+^) cell ratio in WT, HepG2^lv-^ *^FLAP^*, and HepG2^sh-^ *^FLAP^* cells by flow cytometry assay. **(D, E)** Immunoblotting analyses of CYLD and CysLTR2 in WT, HepG2^lv-^ *^FLAP^*, or HepG2^sh-^ *^FLAP^* cells. β-Tubulin was used as loading control. **(F)** Schematic representation of FLAP overexpression results in CYLD attenuation in the HCC canceration process. Error bars, mean value ± SD; Student unpaired *t-*test was used to compare groups, and *p-*value less than 0.05 was considered as statistically significant. ^*^ *p* < 0.05 vs. control, ^**^ *p* < 0.01 vs. control. For each analysis, *n* = 3.

To further confirm the impact of FLAP on CYLD expression, we explored the abundance of CYLD in WT, lv-*FLAP*, and sh-*FLAP* human HCC cell lines according to the scheme of the experimental procedure (**Figure 7A**). Among human HCC cell lines HepG2, Hep3B, and PLC/PRF/5, we validated the favorable nature of FLAP expression in HepG2 cells in response to AAI exposure (**Figure 7B**). We therefore modulated the abundance of FLAP in HepG2 or Hep3B cells to generate HepG2^lv-^ *^FLAP^*, Hep3B ^lv-^ *^FLAP^* or HepG2^sh-^ *^FLAP1^*, HepG2^sh-^ *^FLAP2^*, and Hep3B^sh-^ *^FLAP^* cells. Compared to WT HCC cell lines, HepG2^lv-^ *^Con^* or HepG2^sh-^ *^Con^* cells, we observed that either *FLAP* mRNA or FLAP-positive (FLAP^+^) cell ratio was remarkably high in HepG2^lv-^ *^FLAP^* cells (*p* < 0.01) and decreased in HepG2^sh-^ *^FLAP^* cells (*p* < 0.01) using qRT-PCR (**Supplementary Figure 5**) or flow cytometry assays (**Figure 7C**), indicating that infection or transfection efficiency was significant. Notably, there was an associated reduction of CYLD in HepG2^lv-^ *^FLAP^* cells compared to the WT HCC cell line and HepG2^lv-^ *^Con^* cells (*p* < 0.05), while the expression of CYLD in HepG2^sh-^ *^FLAP2^* cells was significantly higher than WT and HepG2^sh-^ *^Con^* cells (*p* < 0.05), as evidenced by Western blot assay (**Figures 7D**, **E**). Using immunofluorescent staining, similar outcomes appeared in Hep3B cells. The negatively correlated tendency was the same as that in HepG2 cells. The immunofluorescent stained characteristic was quite similar to the liver tissues of AAI-treated canines by immunofluorescence assay (**Supplementary Figure 6**). These results intensively revealed that FLAP was required for negatively regulating CYLD expression in AAI-treated human HCC cells. Given that CysLTR2 was more expressed in the tumor vasculature compared to CysLTR1 (35), we further evaluated CysLTR2 expression in WT, HepG2^lv-^ *^FLAP^*, or HepG2^sh-^ *^FLAP^* HCC cells. Unlike AAI-treated livers of canines, the expression of CysLTR2 did not change in HepG2^lv-^ *^FLAP^* or HepG2^sh-^ *^FLAP^* cells in contrast to WT HCC cells, HepG2^lv-^ *^Con^*, and HepG2^sh-^ *^Con^* cells (**Figures 7D, E**). These data implied that HepG2 cells probably only kept limited expression of CysLTR2, and that its abundance was not significantly affected by FLAP. We suggested that the inherent CysLTR2 was enough to make CysLTs signal transduction, thereby negatively modulating CYLD expression. A schematic representation of the hepatic FLAP/CysLTs cascade contributing to the attenuation of CYLD in AAI-induced hepatic malignant alterations was illustrated in **Figure 7F**.

## Discussion

The prognosis of HCC patients is poor, with only limited treatment options (36). There is an urgent need to seek novel targets for the early diagnostic and preventive strategies of HCC patients. Hepatocarcinogenesis and HCC development are a multistep process, and probably evolve from the inflammation. In the current study, we reveal that all the resection tissues from HCC patients show significant overexpression of FLAP in both malignant lesion and paracarcinoma tissues in contrast to those from donors. Furthermore, FLAP is closely associated with neovascularization (including tumor angiogenesis) and cell proliferation in resection tissues of HCC patients, evidenced by high expression of FLAP or Ki-67, and neovascular localization of CD34 in parallel. Remarkably, the effect of mRNA level of hepatic *FLAP* on MST also displayed a close correlation in HCC patients after surgical resection. Of additional interest is that the high transcription of *FLAP* leads to significant shortening of the MST of HCC patients after surgical resection, indicating that FLAP high expression plays a crucial role in HCC development or recrudescence. Therefore, our findings that hepatic FLAP increased in expression could be a valuable target for both therapeutics and predicting survival of HCC patients after surgical resection.

Since canine and human share a remarkable similarity in cancers (27, 28), the present study takes it as a preponderant animal model for onset and progression research of HCC. Our previous study confirms that hepatic premalignant lesion appears in canines after a 10-day AAI oral administration. It is featured by an increase in *Il6* [interleukin (IL)-6] transcription and supernatant IL-6 level, as well as the overexpression of c-Myc and oncofetal RNA-binding protein Lin28B (29). The premalignant node involves the dysregulation of microRNAs, as well as the activation of IL-6 receptor, p-STAT3, and NF-κB (29). By studying the impact of AAI treatment on 5-LO/CysLTs cascade, the present study further looks into the mechanism that links FLAP to CYLD in canine liver, and we identify a novel candidate driver for hepatic malignant lesion. Following AAI administration of canines, the livers show a significant overexpression of FLAP accompanied by markedly higher CysLTs content. The enhanced hepatic FLAP/CysLTs cascade displays a negative correspondence to CYLD expression and a positive correspondence to its downstream effector JNK phosphorylation. The present study reveals that both the 5-LO/LTB_4_ bypass and the COX/PGE_2_ pathway in AAI-treated livers of canines do not change in expression, indicating that both pathways contribute less to the generation of lipid pro-inflammatory mediators (33). These data convincingly indicate that the more reduced CYLD is associated with increased CysLTs. Most importantly, the data from both HepG2^lv-^ *^FLAP^*, Hep3B^lv-^ *^FLAP^* and HepG2^sh-^ *^FLAP^*, Hep3B^sh-^ *^FLAP^* cells directly confirm the impact of FLAP abundance on CYLD expression after AAI treatment. The *in vitro* results intensively support that hepatic FLAP overexpression is required for suppressing CYLD. Strikingly, our study highlights the fact that the enhanced hepatic FLAP/CysLTs cascade is involved in CYLD suppression. In the liver, CYLD acts as an important regulator of hepatocyte homeostasis (23). In HCC pathogenesis, CYLD is involved in negatively regulating apoptosis, regeneration, NF-κB signaling, and inflammation. Consistent with our previous findings, such as apoptosis, regeneration, NF-κB signaling, and inflammation, as well as an increase in *Il6* transcription and supernatant IL-6 level in AAI-treated livers of canines (29), here, the further evidence that AAI-induced CYLD suppression accompanies the inflammatory cell infiltration around the central veins is quite similar to the features spontaneously appearing in the livers of CYLD-deficient mice (32). Owing to the FLAP overexpression-induced CYLD suppression, we consider that the hepatocyte homeostasis suffers from significant disorders, including all the features described above and the malignant progression in canine livers.

Conversely, the higher abundances of hepatic mGST2 and CysLTR2 do not cooperate with CYLD suppression in donors, although both of them highly express in the HCC tissues, similar to the livers in AAI-treated canines. Furthermore, the overexpression of mGST2 mRNAs does not influence the MST of HCC patients after surgical resection, suggesting that it is at least not involved in inhibiting expression of CYLD. These results intensively indicate that FLAP itself, rather than mGST2 and CysLTR2, is crucially important to HCC development and recrudescence in patients and AAI-induced hepatocarcinogenesis in canines. The compelling findings highlight the possible therapeutic implications of FLAP in human HCC development. Therefore, FLAP overexpression is a noteworthy index parameter running throughout the initiation and termination in liver cancer pathology.

In rat liver, hepatocytes accounted for the highest ability to metabolize and produce CysLTs from the LTA_4_ (37). Using the FLAP as a marker, we confirm that FLAP overexpresses in both hepatocytes and Kupffer cells in canine liver after AAI treatment. Since LTC_4_S only expresses in cells of hematopoietic lineage, the present study does not show that it highly expresses in canine livers, and we believe that majority of CysLTs come from hepatocytes. Given that FLAP aids to catalyze LTA_4_ production, our data reveal that AAI-induced overexpression of FLAP is a major element contributing to CysLTs generation in canine livers. Hepatocyte-derived CysLTs mediate hepatic vascular tone abnormalities in cirrhosis (38). The 5-LO pathway has been indicated to have a role in different cancers (39). CysLTs involve the microenvironment, influencing local risk of malignant transformation (40). To date, the signaling effects of CysLTs/CysLTR2 on oncogenesis and development are controversial. High CysLTR2 expression is correlated with a good prognosis in patients with colorectal cancer (41) and breast cancer (42). However, constitutive activation of CysLTR2 in uveal melanoma acts as an oncogene (43). CysLTR2 expression in stromal cells, rather than tumor cells, is essential for enhanced invasiveness (35). Although high expression of CysLTR2 appears in resection tissues of HCC patients and in AAI-induced premalignant livers of canines, the data from both HepG2^lv-^ *^FLAP^* and HepG2^sh-^ *^FLAP^* cells do not demonstrate the impact of FLAP abundance on CysLTR2 expression. We therefore focus on the hepatic ER stress/CysLTRs axis, since ER stress triggers CysLTRs internalization (14) and ER stress inducers show the ability to reduce CYLD expression (34). Our data demonstrate that AAI treatment triggers the early and late events of ER stress in canine livers, as evidenced by high Grp78 expression and eIF2α phosphorylation, thereby probably promoting CysLTR2 internalization. Of CysLTs, LTC_4_, LTD_4_, and LTE_4_ interact with their own specific receptors. LTC_4_ possesses CysLTR2-specific affinity and potency (16). LTC_4_ is the only intracellular CysLTs; it also outputs and exists in the extracellular space with a very short lifetime. ER stress induces CysLTR2 internalization, making LTC_4_ much easier to activate CysLTR2 intracellularly. Our results confirm that a high FLAP expression is involved in CysLTs generation *in vivo*. Once ER stress triggers CysLTR2 internalization, increased CysLTs have a strong tendency to allow CysLTR2 activation. We consider that it is enough for CysLTs/CysLTR2 signaling transduction to take a local or transient effect on inhibiting CYLD expression in liver cancer pathology. The hepatic ER stress/CysLTR2 axis probably contributes to the FLAP/CysLTs/CYLD/p-JNK signaling transduction *in vivo*.

Nuclear expressions of CysLTRs are potential prognostic indicators of colorectal cancer. High CysLTR2 nuclear expression has the best survival expectancy (41). Our data show that a high FLAP expression along with high nuclear CysLTR2 nuclear expression results in CYLD expression in paracarcinoma tissues, while a high FLAP expression along with high cytosolic CysLTR2 expression leads to CYLD nuclear translocation in malignant lesion. Although our data link CysLTR2 nuclear distribution to CYLD expression in HCC, we cannot confirm whether different styles of CysLTR2 distribution directly affect CYLD expression from the only two HCC patients.

Strikingly, we reveal that FLAP overexpression is correlated with CD34 neovascular localization and high Ki-67 expression in HCC tissues of HBV-infected patients. That means, once hepatic FLAP is detected and inhibited, neovascularization and tumor cell proliferation can probably be inhibited in HCC tissues. Recently, the 5-LO pathway has been highlighted as a promising therapeutic direction in different diseases (8, 9, 44). Our data intensively suggest that despite the fact that the resection tissues from HCC patients or AAI-induced premalignant lesion in canine livers share the same characteristics, FLAP overexpression is parallel to CYLD attenuation. Based on our knowledge, we report here for the first time that FLAP overexpression exists throughout the hepatic premalignant and the terminal HCC period. FLAP inhibition, therefore, may provide the foundation for new therapeutic strategies of HCC patients and could be more significant in prolonging the life span of HCC patients after surgical resection.

## Data availability statement

The raw data supporting the conclusions of this article will be made available by the authors, without undue reservation.

## Ethics statement

Protocols for patient tissue section usage were reviewed and approved by the Ethics Committees of Shulan (Hangzhou) Hospital (№ 20170207). The patients/participants provided their written informed consent to participate in this study. T The protocols were approved by Animal Care and Use Committee of Zhejiang University (№ ZJU2009108012Y), and all procedures were performed in accordance with the institutional guidelines.

## Author contributions

K-kS designed and conducted clinical data and *in vitro* experiments, bioinformatics analysis, statistical analysis, and drafted the manuscript. X-hZ conducted most of the animal experiments and data analysis. CB provided guidance on conception, and reviewed and edited the manuscript. X-hZ. and RH conducted the acquisition of clinical data of HCC patients and donors. D-hZ and J-jT aided in bioinformatics analysis. Y-jL and Y-hZ organized the animal study and acquisition of animal data. L-jL provided guidance on conception, supervised the study for clinic pathological feature and pathological analysis of HCC patients, and granted final approval of the version to be published. All authors contributed to the article and approved the submitted version.

## Funding

This work was supported by the National Key Research and Development Program of China (№ 2019YFC0840609 to K-kS), the National Natural Science Foundation of China (№ 81401707 to K-kS, № 81790630 to L-jL, and № 91229124 to Y-jL), the Zhejiang Provincial Natural Science Foundation (№ LY20H030006 to K-kS), and the Fundamental Research Funds for the Central Universities (Nō 2022ZFJH003 to K-kS and L-jL).

## Acknowledgments

Bang-xin Chen aided Western blotting experiments. Data were eligible for HCC patient survival analysis from the TCGA Research Network.

## Conflict of interest

The authors declare that the research was conducted in the absence of any commercial or financial relationships that could be construed as a potential conflict of interest.

## Publisher’s note

All claims expressed in this article are solely those of the authors and do not necessarily represent those of their affiliated organizations, or those of the publisher, the editors and the reviewers. Any product that may be evaluated in this article, or claim that may be made by its manufacturer, is not guaranteed or endorsed by the publisher.

## Supplementary material

Supplementary Table 1 The information for all the participants donating resection tissues of livers. The characteristics were summarized, concerning etiology, gender, age, and tumor stage.

Supplementary Table 2 Primer sequences (5’-3’) for qRT-PCR.

Supplementary Table 3 sh*FLAP* sequences.

Supplementary Figure 1 Two HCC cases with CYLD expression in resection tissues. One showed CYLD nuclear translocation together with a much higher cytosolic CysLTR2 expression within poorly differentiated HCC lesions (**left panel**), and the other one displayed slight cytosolic CYLD expression together with a much higher nuclear CysLTR2 expression in paracancerous tissue (**right panel**).

Supplementary Figure 2 The inflammatory cell infiltration were evaluated by counting the numbers of inflammatory cells in each section field.

Supplementary Figure 3 The positive and negative controls were set up for immunohistochemical analysis of all observed subjects.

Supplementary Figure 4 Expressions of receptors in LTB4 bypass. Immunoblot analyses of BLTs.β-Actin and GAPDH were used as loading control. Error bars represent the mean value ± SD; A Student unpaired *t* test was used to compare groups, (BLT2 control, *n*=3).

Supplementary Figure 5 Relative expression of *FLAP* mRNAs in Knock-in or Knock-down of *FLAP* in HepG2 cells at the indicating times. Overexpression of *FLAP* in HepG2 cells was achieved by lentiviral infection. Lentivirus-*Control* (lv-*Con*) or lv-*FLAP* (HepG2^lv-^ *^Con^*, HepG2^lv-^ *^FLAP^*) were selected for 3 days. Knockdown of *FLAP* in HepG2 cells was conducted by short hairpin RNA(shRNA). HepG2^lv-^ *^FLAP^* and HepG2^sh-^ *^FLAP^*, and wild type (WT), HepG2^lv-^ *^Con^* or HepG2^sh-^ *^Con^* cells were further incubated with AAI (final concentration 1.25 μM) for another 7 days. Error bars, mean value ± SD; A Student unpaired *t* test was used to compare groups, and a *P* value less than 0.05 was considered statistically significant. ^*^ *P* < 0.05 *vs* control, ^**^ *P*<0.01 *vs* control. For each analysis, *n*=3.

Supplementary Figure 6 Overexpression of FLAP diminishes CYLD expression in AAI-treated Hep3B^lv-^ *^FLAP^* cells. WT HCC cells without AAI treatment were used as control. In the case of AAI treatment, representative photomicrographs of CYLD abundance (red: distribution in cytoplasm, blue: nuclei with DAPI staining) was indicated by immunofluorescence histochemistry. Bar=100 μm, bar=20 μm (magnification).
