# Supplementary material for: Five-lipoxygenase-activating protein-mediated CYLD attenuation is a candidate driver in hepatic malignant lesion
Source: Front Oncol. 2022 Aug 1;12:912881. doi: 10.3389/fonc.2022.912881 (PMC9376481; doi:10.3389/fonc.2022.912881)
Supplement: Supplementary Table 1 — The information for all the participants donating resection tissues of livers. The characteristics were summarized, concerning etiology, gender, age, and tumor stage. [file Table_1.doc]

**Supplementary Table 1 Basic information on the surgical patients and donors under prior informed consent procedure and with written consent of the Human Ethics Committee**

| **Case numbers** | **Sex** | **Age** | **Operation** | **HBV infection** | **Ki-67 Positive rate (%)** | **Pathology diagnosis** |
| --- | --- | --- | --- | --- | --- | --- |
| **68*3 | Male | 56 | Liver donor | No | 1 | Donor liver. |
| **75*3 | Male | 45 | Liver donor | No | <1 | Donor liver. |
| **71*8 | Male | 29 | Liver donor | No | 1 | Donor liver. |
| **65*0 | Male | 35 | Liver donor | No | <1 | Donor liver. |
| **71*2 | Male | 63 | Liver donor | No | 1 | Donor liver. |
| **72*0 | Female | 48 | Liver donor | No | <1 | Donor liver. |
| **72*5 | Male | 46 | Liver donor | No | 1 | Donor liver. |
| **71*8 | Male | 67 | Liver donor | No | 3 | Donor liver. |
| **72*3 | Female | 51 | Liver donor | No | 5 | Donor liver. |
| **68*6 | Female | 52 | Liver donor | No | 2 | Donor liver. |
| **75*4 | Male | 45 | Liver transplantation | No | 1 | M1 moderately differentiated HCC, nodular cirrhosis. |
| **71*7 | Male | 50 | Liver transplantation | Yes | 20 | M2 moderately differentiated HCC, nodular cirrhosis. |
| **75*8 | Male | 54 | Liver transplantation | Yes | 70 | M1 moderately differentiated HCC, diffuse type. |
| **71*9 | Male | 67 | Liver transplantation | Yes | 50 | M0 moderately differentiated HCC, nodular cirrhosis. |
| **76*6 | Male | 73 | Hepatectomy | Yes | 40 | M0 moderately differentiated HCC, Adenoid type. |
| **72*1 | Male | 60 | Hepatectomy | Yes | 50 | M1 moderately differentiated HCC, [nodule type](https://www.baidu.com/link?url=l2HoWp-ZGTwUR3kvCWRxN1f3Ep8Xo0fwYJNE_OXXIdHBqBVCOXKzkxSSQ9wZ9kFueWrtmYHLVoAncAD5Lx7Jaypk7YFml2ZmZK0jmm3fA3il_XF3myv7LrDO_PY-qm6u&wd=&eqid=e5f75aa20000403e000000035a824b82). |
| **72*6 | Male | 46 | Liver transplantation | Yes | 30 | M0 moderately differentiated HCC with necrosis, cirrhosis. |
| **76*5 | Male | 47 | Hepatectomy | Yes | 50 | M0 poor differentiated HCC, nodular cirrhosis. |
| **74*2 | Male | 55 | Liver transplantation | Yes | 70 | M0 moderately differentiated HCC, [multiple nodule type](https://www.baidu.com/link?url=l2HoWp-ZGTwUR3kvCWRxN1f3Ep8Xo0fwYJNE_OXXIdHBqBVCOXKzkxSSQ9wZ9kFueWrtmYHLVoAncAD5Lx7Jaypk7YFml2ZmZK0jmm3fA3il_XF3myv7LrDO_PY-qm6u&wd=&eqid=e5f75aa20000403e000000035a824b82). |
| **72*2 | Male | 60 | Liver transplantation | Yes | 50 | M1 moderately differentiated HCC. |
