# Supplementary material for: Five-lipoxygenase-activating protein-mediated CYLD attenuation is a candidate driver in hepatic malignant lesion
Source: Front Oncol. 2022 Aug 1;12:912881. doi: 10.3389/fonc.2022.912881 (PMC9376481; doi:10.3389/fonc.2022.912881)
Supplement: Supplementary Table 2 — Primer sequences (5’-3’) for qRT-PCR. [file Table_2.doc]

**Supplementary Table 2 Primer sequences (5’-3’) for qRT-PCR**

| **Gene** | | **Primer (5’-3’)** | |
| --- | --- | --- | --- |
| *FLAP* | Sense | | GGCCCTTGTCACCCTCATCAGCG |
|  | Antisense | | AACAGGCCGGCGAAGGACATGAGG |
| *GAPDH* | Sense | | AGAAGGCTGGGGCTCATTTG |
|  | Antisense | | AGGGGCCATCCACAGTCTTC |
| *Lta4h* | Sense | | TTAGTCAGTGCCAGGCCATC |
|  | Antisense | | TCATACTGGCCCCAGACGTA |
| *Gapdh* | Sense | | GGGGCCATCCACAGTCTTCT |
|  | Antisense | | GCCAAAAGGGTCATCATCTC |
