# Supplementary material for: Five-lipoxygenase-activating protein-mediated CYLD attenuation is a candidate driver in hepatic malignant lesion
Source: Front Oncol. 2022 Aug 1;12:912881. doi: 10.3389/fonc.2022.912881 (PMC9376481; doi:10.3389/fonc.2022.912881)
Supplement: Supplementary Table 3 — shFLAP sequences. [file Table_3.doc]

**Supplementary Table 3 sh*FLAP* sequences**

| **Name** | **Target position** | **Target sequence**  **(21nt target + 2nt overhang)** |
| --- | --- | --- |
| sh*FLAP*-1 | 69-91 | TGGATTCTTTGCCCATAAAGTGG |
| sh*FLAP*-2 | 384-406 | TGGCATATTCAACTATTACCTCA |
