# Supplementary figures and images for: Five-lipoxygenase-activating protein-mediated CYLD attenuation is a candidate driver in hepatic malignant lesion
Source: Front Oncol. 2022 Aug 1;12:912881. doi: 10.3389/fonc.2022.912881 (PMC9376481; doi:10.3389/fonc.2022.912881)

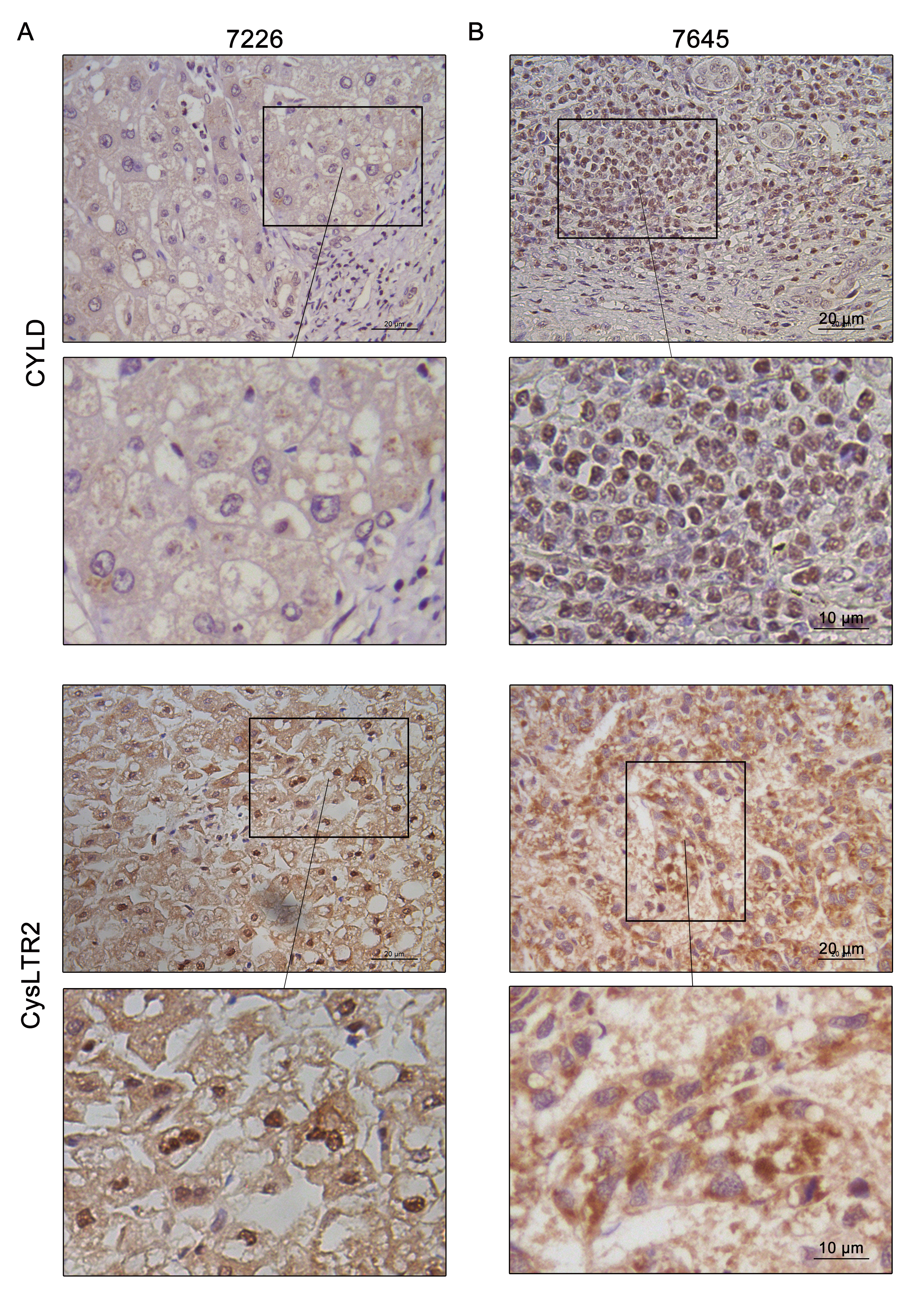

Supplement: Supplementary Figure 1 — Two HCC cases with CYLD expression in resection tissues. One showed CYLD nuclear translocation together with a much higher cytosolic CysLTR2 expression within poorly differentiated HCC lesions (left panel), and the other one displayed slight cytosolic CYLD expression together with a much higher nuclear CysLTR2 expression in paracancerous tissue (right panel). [file Image_1.tif]

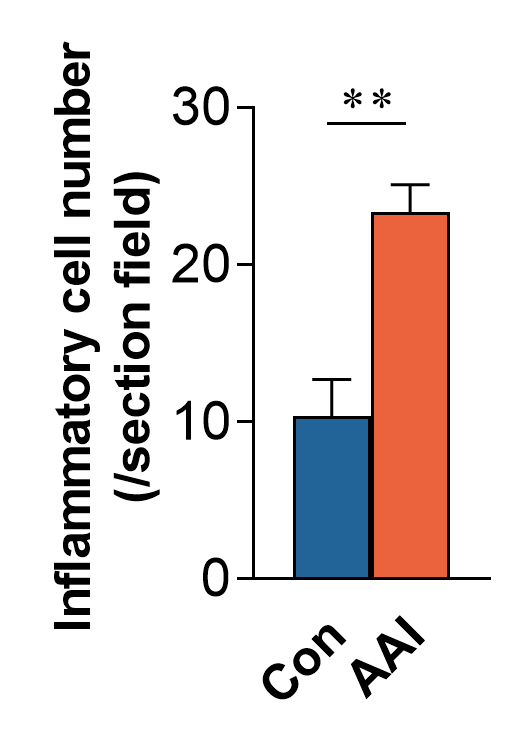

Supplement: Supplementary Figure 2 — The inflammatory cell infiltration were evaluated by counting the numbers of inflammatory cells in each section field. [file Image_2.tif]

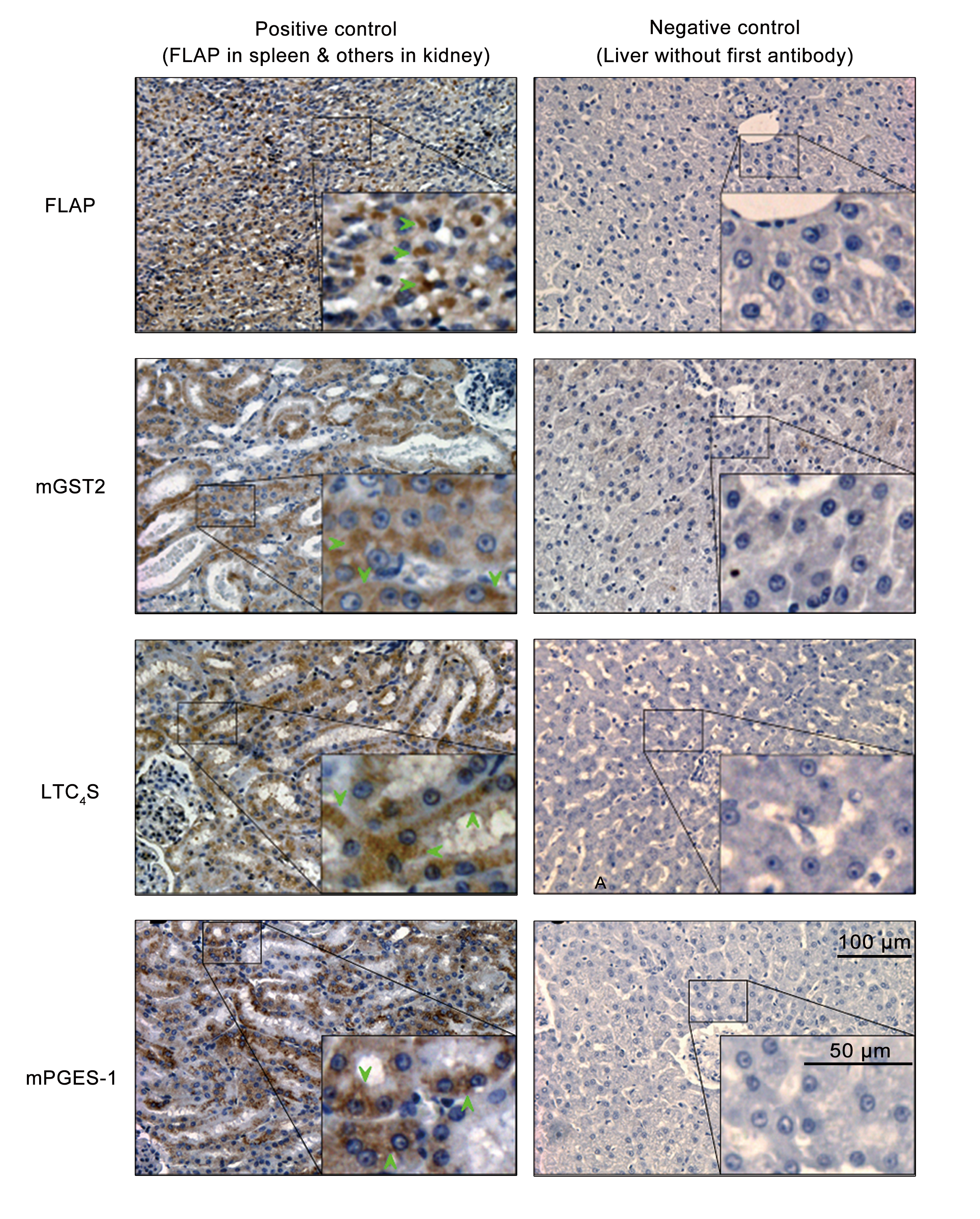

Supplement: Supplementary Figure 3 — The positive and negative controls were set up for immunohistochemical analysis of all observed subjects. [file Image_3.tif]

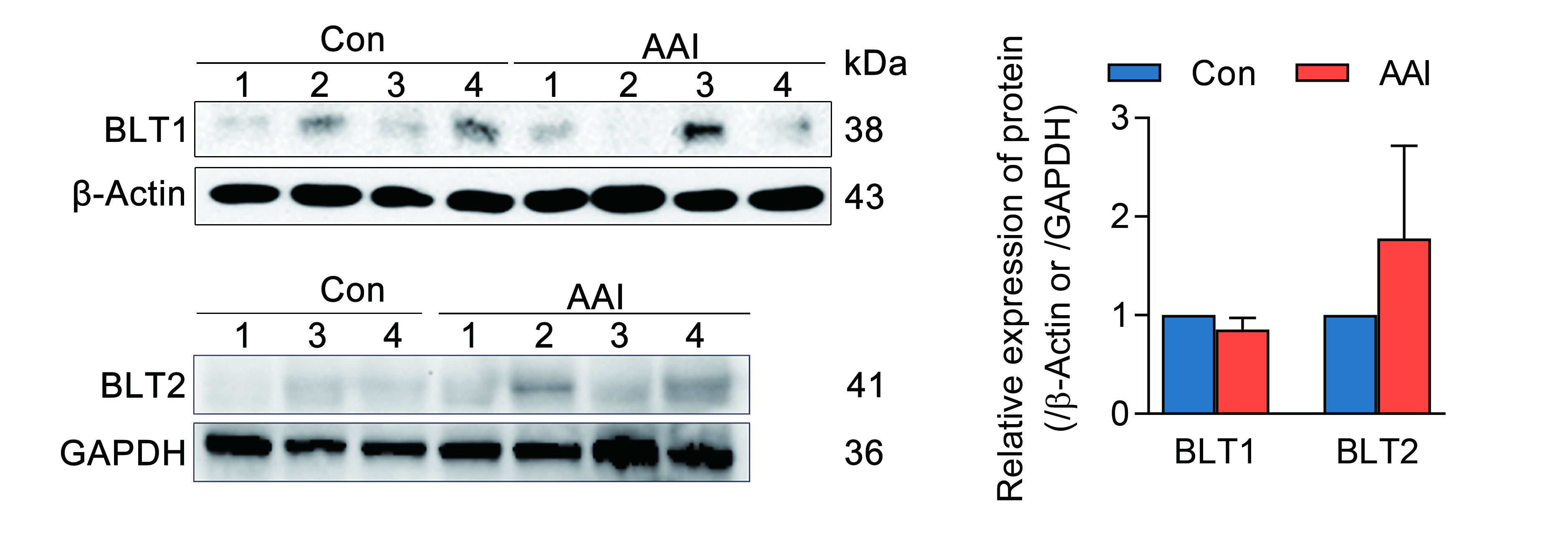

Supplement: Supplementary Figure 4 — Expressions of receptors in LTB4 bypass. Immunoblot analyses of BLTs.β-Actin and GAPDH were used as loading control. Error bars represent the mean value ± SD; A Student unpaired t test was used to compare groups, (BLT2 control, n=3). [file Image_4.tif]

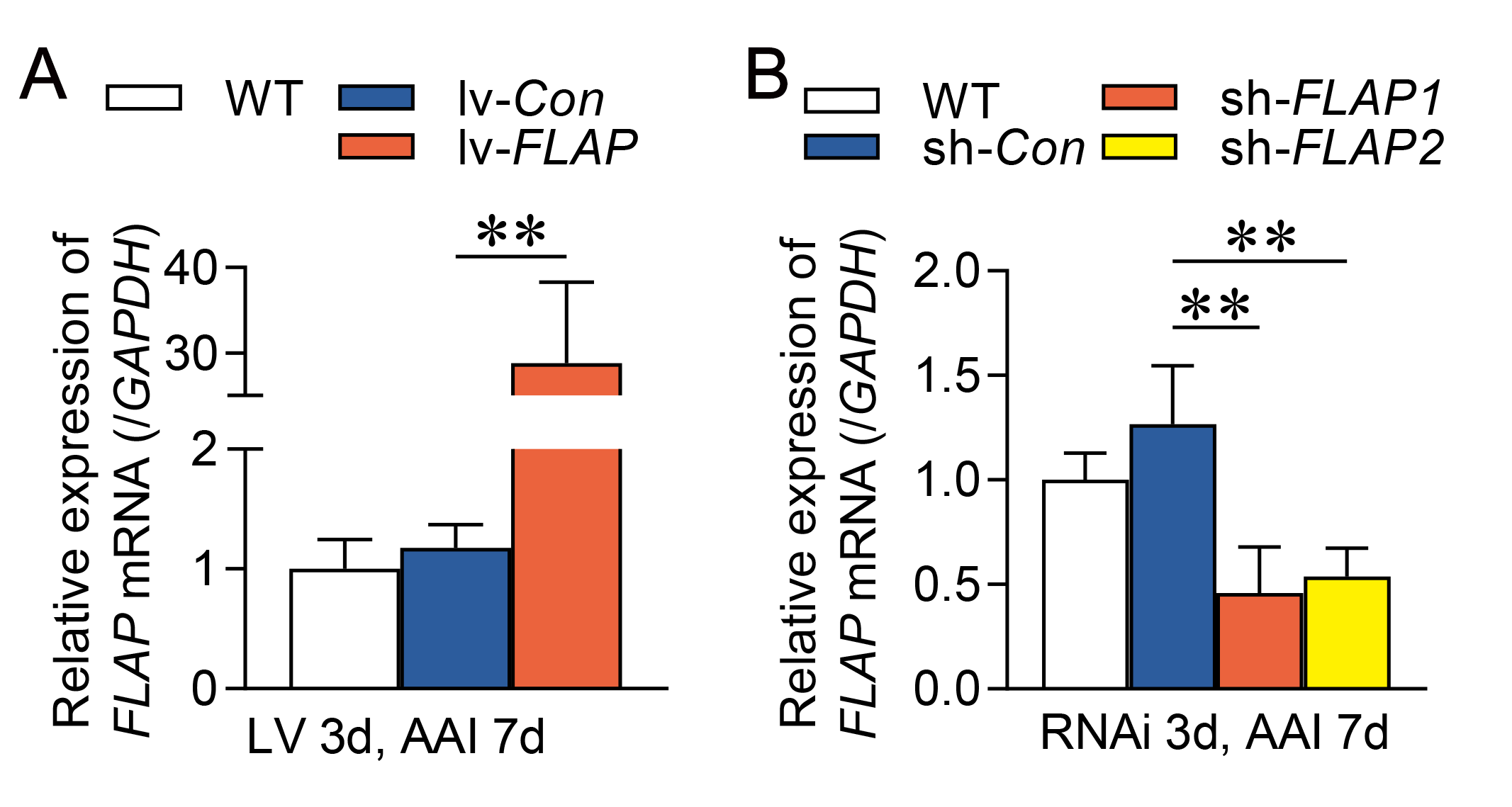

Supplement: Supplementary Figure 5 — Relative expression of FLAP mRNAs in Knock-in or Knock-down of FLAP in HepG2 cells at the indicating times. Overexpression of FLAP in HepG2 cells was achieved by lentiviral infection. Lentivirus-Control (lv-Con) or lv-FLAP (HepG2lv- Con , HepG2lv- FLAP ) were selected for 3 days. Knockdown of FLAP in HepG2 cells was conducted by short hairpin RNA(shRNA). HepG2lv- FLAP and HepG2sh- FLAP , and wild type (WT), HepG2lv- Con or HepG2sh- Con cells were further incubated with AAI (final concentration 1.25 μM) for another 7 days. Error bars, mean value ± SD; A Student unpaired t test was used to compare groups, and a P value less than 0.05 was considered statistically significant. * P < 0.05 vs control, ** P<0.01 vs control. For each analysis, n=3. [file Image_5.tif]

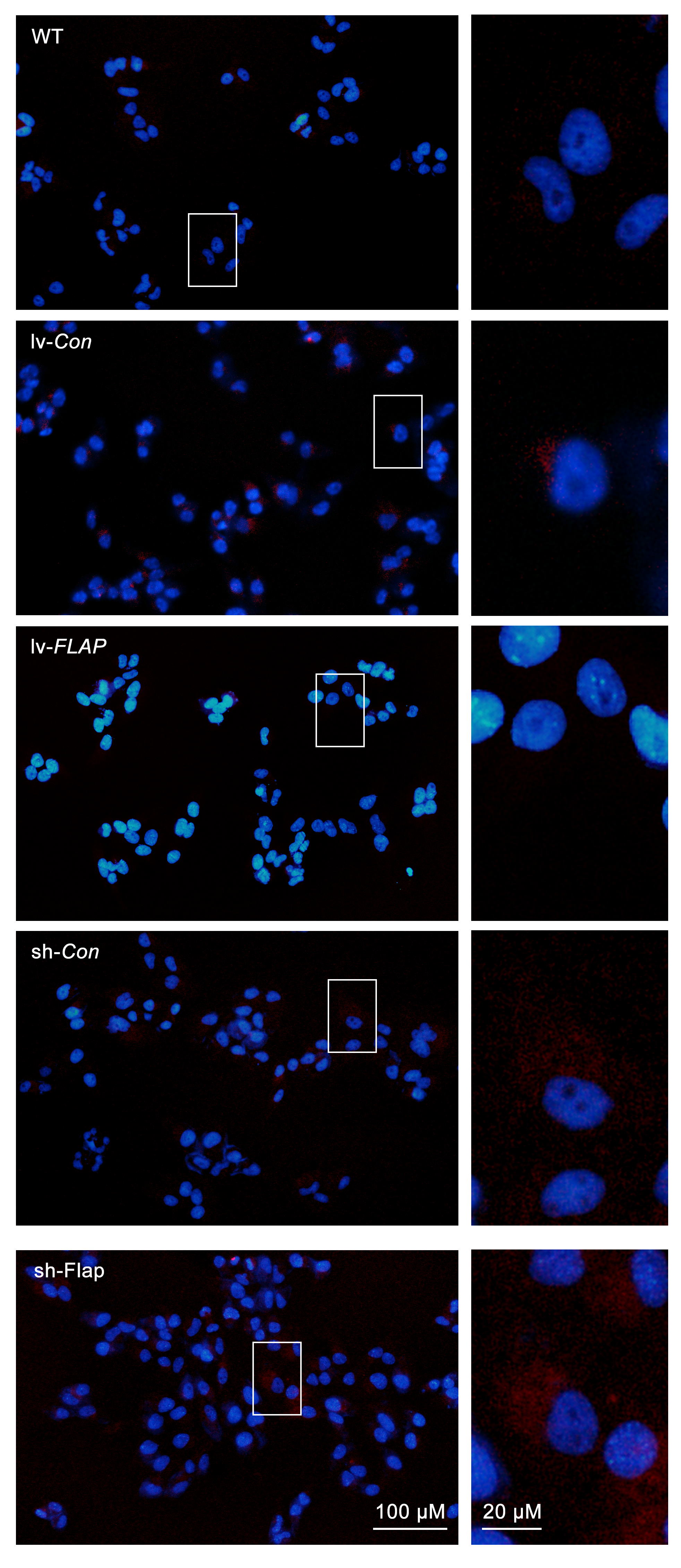

Supplement: Supplementary Figure 6 — Overexpression of FLAP diminishes CYLD expression in AAI-treated Hep3Blv- FLAP cells. WT HCC cells without AAI treatment were used as control. In the case of AAI treatment, representative photomicrographs of CYLD abundance (red: distribution in cytoplasm, blue: nuclei with DAPI staining) was indicated by immunofluorescence histochemistry. Bar=100 μm, bar=20 μm (magnification). [file Image_6.tif]
